# Watershed prioritization of Kailali district through morphometric parameters and landuse/landcover datasets using GIS

**DOI:** 10.1016/j.heliyon.2023.e16489

**Published:** 2023-05-23

**Authors:** Susil Ojha, Lila Puri, Suraj Prasad Bist, Arjun Prasad Bastola, Bishwabandhu Acharya

**Affiliations:** Institute of Forestry Pokhara Campus, Tribhuvan University, Nepal

**Keywords:** Compound value, Principal component analysis, Soil erosion, Weighted sum analysis

## Abstract

Watershed prioritization is considered an important tool for soil and watershed management. This study focuses on the watershed prioritization of the Kailali district in terms of soil erosion, considering morphometric parameters and land use/landcover (LULC) datasets using GIS. ALOS DEM of 30 *m* resolution was used to delineate sub-watersheds and calculate linear, areal, and relief morphometric parameters. Similarly, Esri LULC 2021 (Sentinel-2 imagery at 10 *m* resolution) was used to calculate LULC parameters. An integrated approach of Principal Component Analysis (PCA) and Weighted Sum Analysis (WSA) was used for prioritization. PCA was used to reduce selected parameters, calculate the correlation matrix, and define the significant parameters. WSA was used to define weightage value, and Compound Value (CV) was calculated for the ranking of sub-watersheds. 22 sub-watersheds with at least 3rd order stream and 15 parameters were selected for prioritization. PCA integrated with WSA was found to be effective for prioritization. The findings showed that about 61.58% of the watershed area is in the high-priority category, suggesting those areas are at a higher risk of erosion. Therefore, different land rehabilitation programs and bioengineering techniques should be focused on the sub-watershed of high-priority categories followed by medium and low-priority categories to control further soil erosion. The adopted methodology of prioritization can also be performed for multi-hazard mapping.

## 1 Introduction

Nepal has fragile geography and diverse topography and climatic conditions. It is prone to natural disasters such as floods and landslides making it the 20th top most disaster-prone country in the world [1]. Chure-originated rivers like Mohana and its tributaries flow across the Kailali district. The flow of these rivers is dependent on monsoon precipitation and their flow level depletes significantly during the non-monsoon period [2]. The short duration of intense rainfall in these areas can cause sudden flash floods in the monsoon season which carry a huge volume of rocks, and debris [3] which deteriorate the soil and watershed health [4].

It is not feasible for the management of a watershed as a whole. So, watershed prioritization is necessary for the effective management of watersheds. Watershed prioritization is the process of identification and ranking of environmentally degraded Sub-watershed for the prevention of soil erosion, management of floods, drought, and application of varying levels of conservation treatment [5,6]. The prioritization uses the key issue of the watershed problem, including erosion, as the principal consideration [7].

Various studies [[6], [7], [8], [9], [10], [11]] have been conducted using morphometric parameters and land use and landcover datasets to define priority level of a watershed using GIS technology and tools. Morphometric parameters provide an accurate quantitative description of a basin geometry [12]. It is the mathematical measurement of the configuration of the surface, shape, and dimension of the earth's landforms and its analysis [13] that provide knowledge about the characteristics of the watershed and the hydrological process within it. The morphometric parameters can be classified into linear, areal, and relief aspects. The linear aspect consists of bifurcation ratio, stream length, mean stream length, and stream length ratio; the areal aspect consists of stream frequency, drainage density, drainage texture, constant of channel maintenance, infiltration number, length of overland flow, and shape parameters such as form factor, elongation ratio, circularity ratio, and gravelius compactness coefficient. Similarly, the relief aspect consists of relief ratio, ruggedness number, and dissection index [7,9,10,14]. These parameters can be obtained and computed using DEM data with GIS software and different mathematical formula suggested by Refs. [12,[15], [16], [17], [18], [19], [20]]. Similarly, land use and land cover have also been considered important components for prioritization as they influence the hydrological process by influencing soil erosion and other factors [8,21,22].

Different analysis techniques like Principal Component Analysis [9], Weighted Sum Analysis [10], Fuzzy Analytic Hierarchy Process [23], and Multi-Criteria Ranking Method [5,24] have been applied for analysis in the prioritization. Integration of Principal Component Analysis (PCA) and Weighted Sum Analysis (WSA) is robust enough to define significant and more effective parameters for the watershed prioritization [7]. PCA was used to define the significant parameters whereas WSA was to determine the weights for significant parameters and the compound values for priority ranking.

So far, no research study has been conducted in the study area to analyze and prioritize the watershed. As prioritization of watersheds for their relative stability in terms of land erosion is an effective tool for soil and watershed management, a study for the prioritization of watersheds in the study area is necessary. This research is an attempt to prioritize the watersheds of the Kailali district using morphometric parameters and land use/landcover in terms of soil erosion through the integration of PCA and WSA approach for soil and water conservation practices. The study provides baseline information for making informed decisions toward effective soil and watershed management.

## 2 Materials and methods

### 2.1 Study area

**Fig. 1:**
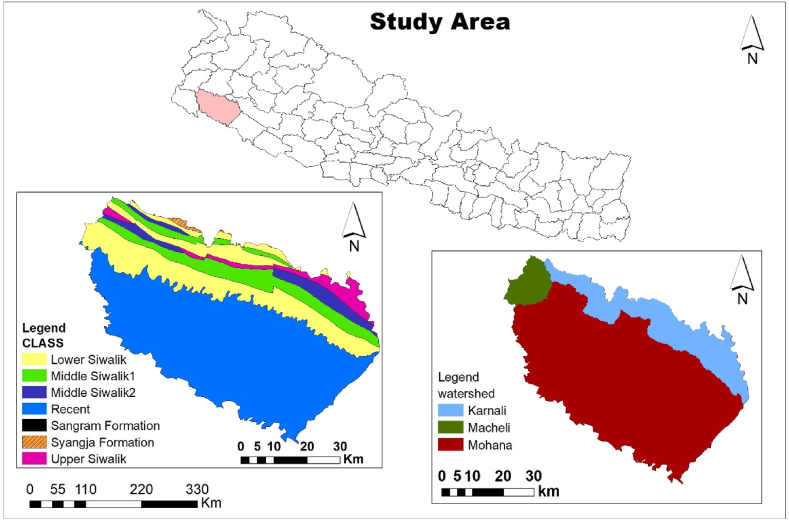
Study area map.

The Kailali district lies between 28°22' - 29°05′ North and 80°30′-81°18′ East with an altitude ranging from 135 *m* to 1967 *m*. It has an area of 3235sq. Km. About 41.48% of kailali is covered by chure hills (Fig. 1). This region is made up of sandstone, greywacke, and arkose. The major rivers of kailali are karnali, mohana, khutiya, patharaiya, roar, donda, shivganga, gaurishankar, kandra, manahara, godawari, likma and gulara. Kailali district is occupied by three watersheds Mohana (76.22%), Macheli (4.92%), and Karnali (18.86%).

### 2.2 Data preparation

#### 2.2.1 Watershed delineations

**Fig. 2:**
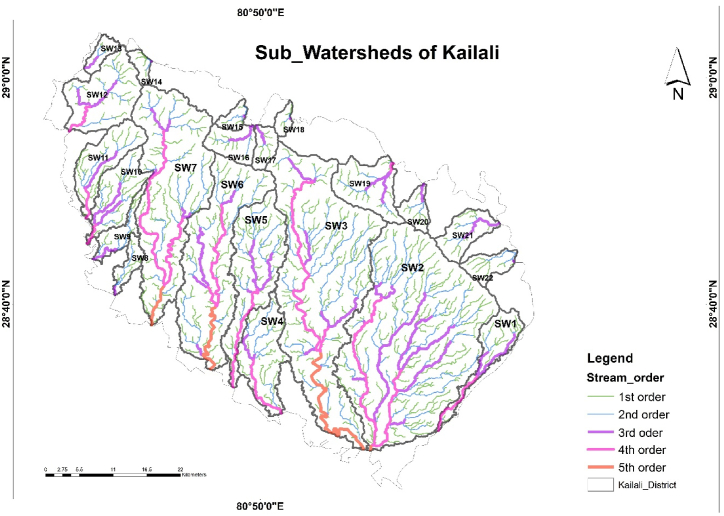
Selected Sub-Watersheds of Kailali district.

First of all, DEM preparation was done for sub-watershed delineation using O'Callaghan and Mark model i.e., fill the sink and determine the flow direction raster using the D8 flow direction algorithm. Sub-watersheds were created using basin functions under hydrology tools in spatial analyst tools and vectorized [25]. For the extraction of the drainage network; a flow accumulation raster was created using a flow direction raster as an input. After a process of iterative and careful visual comparison between drainage networks from google earth online and streams generated with various cell thresholds using a raster calculator, a value of 1000 was found appropriate and selected for the study. The extracted drainage network was ordered based on Strahler's classification through the stream order function. The Sub-watersheds with at least 3rd order streams were selected for further analysis in the study. 22 Sub-watersheds were selected as it meets the required criteria as shown in Fig. 2. Morphometric analysis of the selected 22 Sub-watersheds was done using ArcGIS.

#### 2.2.2 Landuse and landcover parameter

**Fig. 3:**
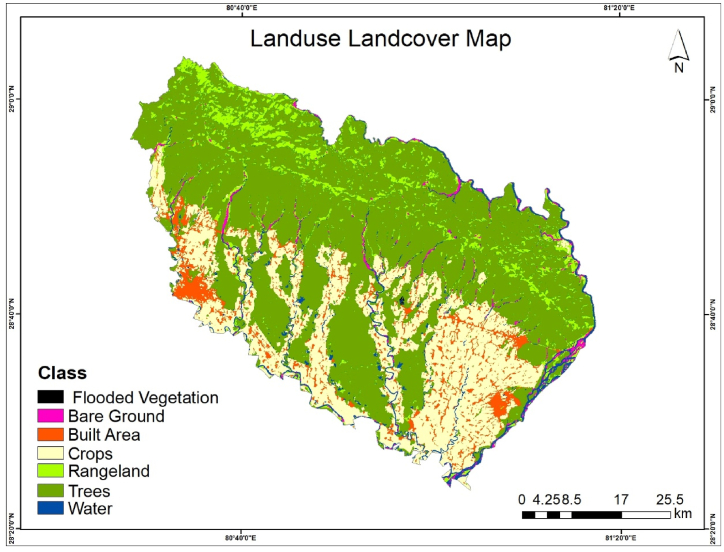
Lulc map of kailali district.

Esri 2021 Lulc map was clipped and projected to WGS 1984 UTM zone 44 N. The Lulc of the study area is shown in Fig. 3.

**Fig. 4:**
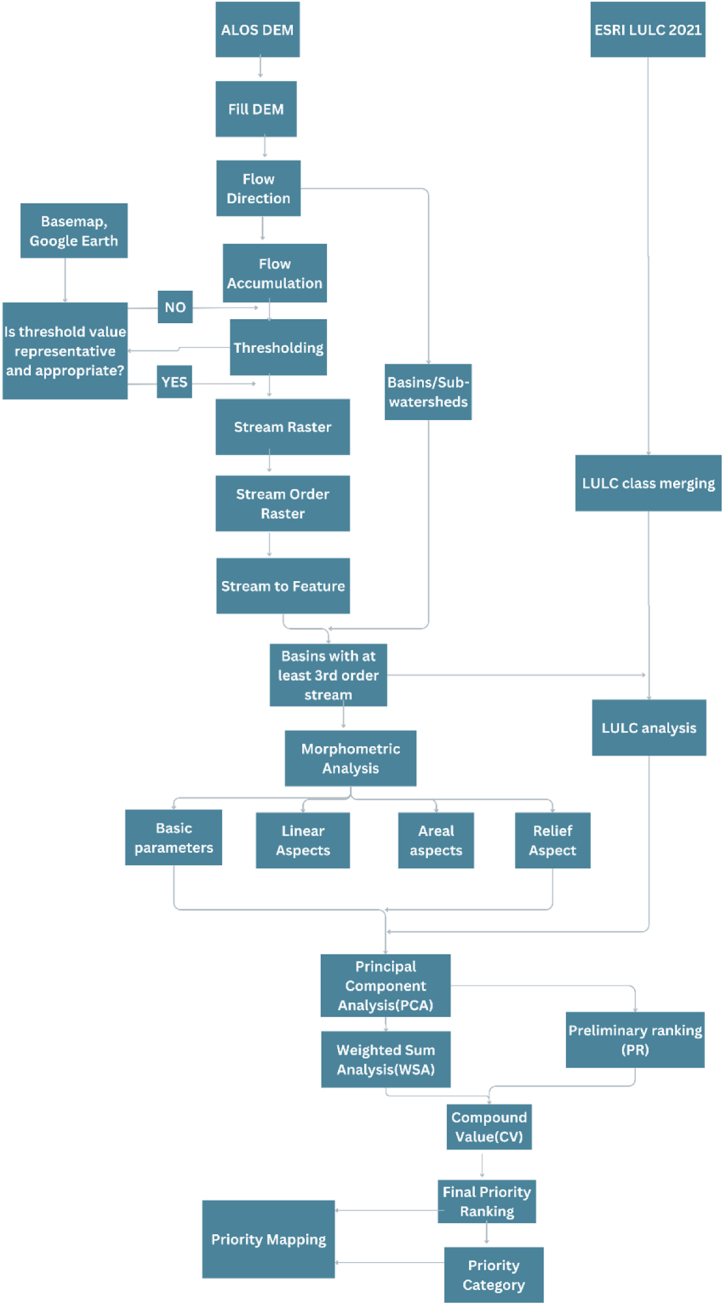
The methodological framework for watershed prioritization.

Lulc raster map was converted to vector polygon and Lulc for each selected Sub-watershed was extracted. The Lulc classes with a similar relationship with erodibility were merged and two Lulc classes were defined to have a strong influence on the hydrological process in the watershed: trees (trees + rangelands) and crops (crops + barren lands + built-up). Flooded vegetation classes were excluded as they may not affect the prioritizations significantly due to their small areas. Fig. 4 Summarizes the entire methodology.

#### 2.2.3 .Morphometric parameters calculations

Different basic, linear, areal, and relief morphometric parameters were calculated using the relation presented in Table 1.

**Table 1:** Formula for calculation of morphometric parameters.

Morphometric Parameters	Formula	References
Linear aspect:		
1. Basin length (Lb)	GIS (km)	[15]
2. Perimeter of the basin (P)	GIS operation (km)	[16]
3. Stream order (u)	Hierarchical order of streams	[17]
4. Stream number (Nu)	Sum of streams for all order (N_u_ = N_1_+N_2_+ … N_n_)	[16]
5. Bifurcation ratio (Rb)	$Rb=N/N_{u+1}$ where, N_u+1_ = total stream in the next order	[15]
6. Stream length (Lu)	Linear measurement of each stream/GIS (km)	[16]
7. Mean stream length (Lsm)	Lsm = Lu/Nu (km)	[12]
8. Stream length ratio (Rl):	Rl = lu/l_u-1_, where lu = total stream length of an order u, l_u-1_ = stream length of next lower order	[15]
9. Mean bifurcation ratio (Rbm)	Average of bifurcation ratio of all orders	[12]
Areal aspect		
10.1. Basin area (A)	GIS operation (km^2)	[16]
10.2. Watershed shape:		
10.3. Form factor (Rf)	$Rf=A/{Lb}^{2}$	[16]
10.4. Elongation ratio (Re)	$Re=2/L_{b}\sqrt{A/\pi }$	[15]
10.5. Circularity ratio (Rc)	$Rc=4*pi*A/P^{2}$	[18]
10.6. Compactness coefficient (*Cc*)	$Cc=P/2\sqrt{\pi A}$	[16]
11. Stream frequency (Fs)	$Fs=N_{u}/A$	[16]
12. Drainage density (Dd)	$Dd=L_{u}/A$	[26]
13. Drainage texture (Dt)	$Dt=N_{u}/P$	[26]
14. Constant of Channel Maintenance (Ccm)	$Ccm=1/D_{d}$	[15]
15. Infiltration number (If)	If=Fs*Dd	[19]
16. Length of overland flow (Lo)	$Lo=1/{2D}_{d}$	[16]
Relief aspect		
17. Max. Relief (Hmax) &Min. Relief (Hmin)	DEM 30 *m* resolution (GIS)	–
18. Basin Relief (Br)	$H_{\max}-H_{\min}$ (m)	[15]
19. Relief ratio (Rr)	$Rr=B_{r}/L_{b}*1000$	[15]
20. Ruggedness number (Rn)	$Rn=Dd*B_{r}/1000$	[26]
21. Dissection index (Di)	$DI=B_{r}/H_{\max}$	[20]

**Source:** [7,10,14].

### 2.3 Data analysis

#### 2.3.1 Principal Component Analysis (PCA) and Weighted Sum Analysis (WSA)

PCA was used for reduction in the selected parameters to find the correlation matrix and to obtain Principal components (PCs) as well to get the most significant parameters. Dimension reduction tool in SPSS software was used for PCA calculations. Initially, KMO and Bartlett's Test was performed to measure sampling adequacy for overall datasets and check if data is suitable for data reduction for the applicability of PCA. Then correlation matrix, first-factor loading matrix, and rotated factor loading matrix were used for further analysis. Eigenvalue>1 was used to select PCs based on the Kaiser criterion and Varimax rotation was used as a rotated factor loading [7]. The significant parameters were calculated based on rotated factor loading. The morphometric parameters and LULC parameters of each sub-watershed were selected for PCA. Preliminary ranking of significant parameters (PRsp)was done on the relationship to the erodibility such that Fs, Dd, Dt, If, and Lo have a direct relationship with erodibility i.e., a higher value indicates more erodibility and higher ranking and vice versa whereas shape parameters and Ccm have indirect relationship i.e., a low value indicates high erosion and higher ranking and vice versa [7,9,10]. Similarly, crops have a direct relationship and trees have an indirect relationship with erodibility [7].

A weighted sum approach was applied to those significant parameters. The weighted value of significant parameters (W_sp_) was calculated using the correlation matrix of significant parameters as explained in equation (1):

(1) Wsp=sumofcorrelationmatrix/totalofcorrelation

The final priority ranking was done based on compound value (CV) such that the lowest value CV was given 1 priority ranking, the second-lowest value was given 2 priority ranking, and so on for all Sub-watershed. CV was calculated using equation (2):

(2) CV=∑(PRsp*Wsp)

These Sub-watersheds were then classified into high, medium, and low categories of risk of erosion for priority mapping.

## 3 Results and discussion

### 3.1 Morphometric and landuse landcover parameters

The morphometric parameter values of selected sub-watersheds are shown in Table 2, Table 3, Table 4.

1. Linear parameter:

**Table 2:** Basic parameters.

sub-watershed	A (km^2^)	P (km)	Lb (km)	MaxElev(m)	MinElev(m)	Basin Relief (Br)
sw1	57.39	70.16	23.4	1153	148	1005
sw2	615.68	136.5	37.73	1552	137	1415
sw3	512.89	173.2	52.14	1771	138	1633
sw4	100.75	69.62	20.36	205	148	57
sw5	157.86	100.3	32.98	1386	152	1234
sw6	267.59	117.8	35.29	1717	155	1562
sw7	327.07	124.6	39.23	1962	165	1797
sw8	21.62	33.84	10.64	222	172	50
sw9	30.95	48.75	15.37	312	181	131
sw10	75.17	56.75	18.35	1295	183	1112
sw11	76.74	54.13	17.58	1787	187	1600
sw12	113.67	54.49	16.63	1965	283	1682
sw13	16.89	22.54	7.06	1559	738	821
sw14	12.99	18.39	4.36	1967	929	1038
sw15	14.26	18.26	5.1	1937	628	1309
sw16	39.71	29.16	8.92	1956	586	1370
sw17	22.92	21.49	7.39	1668	584	1084
sw18	11.15	15.97	3.53	1597	428	1169
sw19	78.4	45.24	11.03	1722	286	1436
sw20	24.47	24.17	8.21	1480	272	1208
sw21	47.04	33.98	11.02	1543	215	1328
sw22	29.85	28.14	9.03	1540	211	1329

**Table 3:** Linear parameters.

S·N	parameter	Stream order					total	S·N.	parameter	Stream Order					total
		1st	2nd	3rd	4th	5th				1st	2nd	3rd	4th	5th	
Sw1	Lu	29.96	7.11	16.07	12.61		65.75	Sw12	Lu	38.54	21.35	12.54	7.31		79.73
	Rl		0.24	2.26	0.78				Rl		0.55	0.59	0.58		
	Lsm	2.00	1.02	3.21	6.31				Lsm	1.01	0.97	1.14	1.83		
	Nu	15.00	7.00	5.00	2.00		29.00		Nu	38.00	22.00	11.00	4.00		75.00
	Rb	2.14	1.40	2.50					Rb	1.73	2.00	2.75			
Sw2	Lu	340.33	189.05	87.98	49.63	0.95	667.94	Sw13	Lu	4.91	3.59	3.38			11.88
	Rl		0.56	0.47	0.56	0.02			Rl		0.73	0.94			
	Lsm	1.72	1.78	1.69	1.38	0.95			Lsm	0.61	1.20	1.13			
	Nu	198.00	106.00	52.00	36.00	1.00	393.00		Nu	8.00	3.00	3.00			14.00
	Rb	1.87	2.04	1.44	36.00				Rb	2.67	1.00				
Sw3	Lu	212.99	157.65	29.95	45.64	30.56	476.79	Sw14	Lu	7.39	2.58	0.18			10.15
	Rl		0.74	0.19	1.52	0.67			Rl		0.35	0.07			
	Lsm	1.28	1.77	1.20	1.30	1.91			Lsm	1.48	0.86	0.18			
	Nu	167.00	89.00	25.00	35.00	16.00	332.00		Nu	5.00	3.00	1.00			9.00
	Rb	1.88	3.56	0.71	2.19				Rb	1.67	3.00				
Sw4	Lu	53.72	31.94	6.53	12.99		105.18	Sw15	Lu	4.67	6.21	0.50			11.38
	Rl		0.59	0.20	1.99				Rl		1.33	0.08			
	Lsm	1.99	2.46	1.31	1.62				Lsm	0.78	2.07	0.25			
	Nu	27.00	13.00	5.00	8.00		53.00		Nu	6.00	3.00	2.00			11.00
	Rb	2.08	2.60	0.63					Rb	2.00	1.50				
Sw5	Lu	79.50	42.32	20.78	20.46		163.06	Sw16	Lu	15.40	7.15	6.18			28.73
	Rl		0.53	0.49	0.98				Rl		0.46	0.86			
	Lsm	1.50	1.51	1.48	2.27				Lsm	1.10	1.02	1.03			
	Nu	53.00	28.00	14.00	9.00		104.0		Nu	14.00	7.00	6.00			27.00
	Rb	1.89	2.00	1.56					Rb	2.00	1.17				
Sw6	Lu	115.87	59.80	24.67	25.42	15.81	241.57	Sw17	Lu	11.91	1.11	4.10			17.12
	Rl		0.52	0.41	1.03	0.62			Rl		0.09	3.71			
	Lsm	1.40	1.42	2.24	1.41	1.44			Lsm	1.70	0.37	1.37			
	Nu	83.00	42.00	11.00	18.00	11.00	165.0		Nu	7.00	3.00	3.00			13.00
	Rb	1.98	3.82	0.61	1.64				Rb	2.33	1.00				
Sw7	Lu	142.17	77.26	13.55	50.66	7.60	291.24	Sw18	Lu	5.77	1.82	0.42			8.01
	Rl		0.54	0.18	3.74	0.15			Rl		0.32	0.23			
	Lsm	1.38	1.43	1.13	1.88	0.84			Lsm	1.15	0.91	0.21			
	Nu	103.0	54.00	12.00	27.00	9.00	205.00		Nu	5.00	2.00	2.00			9.00
	Rb	1.91	4.50	0.44	3.00				Rb	2.50	1.00				
Sw8	Lu	11.15	10.13	1.75			23.03	Sw19	Lu	28.46	13.84	12.21	1.95		56.45
	Rl		0.91	0.17					Rl		0.49	0.88	0.16		
	Lsm	1.39	1.69	1.75					Lsm	0.95	0.77	1.53	0.65		
	Nu	8.00	6.00	1.00			15.00		Nu	30.00	18.00	8.00	3.00		59.00
	Rb	1.33	6.00						Rb	1.67	2.25	2.67			
Sw9	Lu	13.77	13.93	5.97			33.67	Sw20	Lu	11.25	5.31	3.73			20.28
	Rl		1.01	0.43					Rl		0.47	0.70			
	Lsm	1.38	2.32	1.99					Lsm	1.61	1.33	1.86			
	Nu	10.00	6.00	3.00			19.00		Nu	7.00	4.00	2.00			13.00
	Rb	1.67	2.00						Rb	1.75	2.00				
Sw10	Lu	41.43	19.31	17.20	4.43		82.37	Sw21	Lu	17.50	8.90	9.63			36.03
	Rl		0.47	0.89	0.26				Rl		0.51	1.08			
	Lsm	1.80	1.49	2.46	2.22				Lsm	1.17	1.78	1.07			
	Nu	23.00	13.00	7.00	2.00		45.00		Nu	15.00	5.00	9.00			29.00
	Rb	1.77	1.86	3.50					Rb	3.00	0.56				
Sw11	Lu	42.48	17.31	9.78	5.70		75.27	Sw22	Lu	16.74	9.33	0.45			26.52
	Rl		0.41	0.57	0.58				Rl		0.56	0.05			
	Lsm	2.50	2.16	4.89	0.95				Lsm	2.39	2.33	0.23			
	Nu	17.00	8.00	2.00	6.00		33.00		Nu	7.00	4.00	2.00			13.00
	Rb	2.13	4.00	0.33					Rb	1.75	2.00

**Table 4:** Selected parameters for prioritization.

sub-watershed	linear aspect	Areal aspect										Relief parameter			lulc parameter	
	Rbm	Rf	Re	Rc	*Cc*	Fs	Dd	Dt^a^	Ccm	If	Lo	Rr	DI	Rn	trees%	crops%
Sw1	2.01	0.10	0.37	0.15	2.61	0.51	1.15	0.41	0.87	0.58	0.44	0.04	0.87	1.15	80.25	11.62
Sw2	10.34	0.43	0.74	0.42	1.55	0.64	1.08	2.88	0.92	0.69	0.46	0.04	0.91	1.54	40.34	59.26
Sw3	2.08	0.19	0.49	0.21	2.16	0.65	0.93	1.92	1.08	0.60	0.54	0.03	0.92	1.52	66.93	31.52
Sw4	1.77	0.24	0.56	0.26	1.96	0.53	1.04	0.76	0.96	0.55	0.48	0.00	0.28	0.06	64.84	34.15
Sw5	1.82	0.15	0.43	0.20	2.25	0.66	1.03	1.04	0.97	0.68	0.48	0.04	0.89	1.27	60.94	38.45
Sw6	2.01	0.21	0.52	0.24	2.03	0.62	0.90	1.40	1.11	0.56	0.55	0.04	0.91	1.41	69.59	29.24
Sw7	2.46	0.21	0.52	0.26	1.94	0.63	0.89	1.65	1.12	0.56	0.56	0.05	0.92	1.60	84.19	14.91
Sw8	3.67	0.19	0.49	0.24	2.05	0.69	1.07	0.44	0.94	0.74	0.47	0.00	0.23	0.05	33.98	65.33
Sw9	1.83	0.13	0.41	0.16	2.47	0.61	1.09	0.39	0.92	0.67	0.46	0.01	0.42	0.14	20.02	79.90
Sw10	2.38	0.22	0.53	0.29	1.85	0.60	1.10	0.79	0.91	0.66	0.46	0.06	0.86	1.22	58.08	41.58
Sw11	2.15	0.25	0.56	0.33	1.74	0.43	0.98	0.61	1.02	0.42	0.51	0.09	0.90	1.57	81.99	17.53
Sw12	2.16	0.41	0.72	0.48	1.44	0.66	0.70	1.38	1.43	0.46	0.71	0.10	0.86	1.18	98.98	0.86
Sw13	1.83	0.34	0.66	0.42	1.55	0.83	0.70	0.62	1.42	0.58	0.71	0.12	0.53	0.58	99.61	0.39
Sw14	2.33	0.68	0.93	0.48	1.44	0.69	0.78	0.49	1.28	0.54	0.64	0.24	0.53	0.81	98.17	1.53
Sw15	1.75	0.55	0.84	0.54	1.36	0.77	0.80	0.60	1.25	0.62	0.63	0.26	0.68	1.04	99.29	0.20
Sw16	1.58	0.50	0.80	0.59	1.31	0.68	0.72	0.93	1.38	0.49	0.69	0.15	0.70	0.99	99.85	0.09
Sw17	1.67	0.42	0.73	0.62	1.27	0.57	0.75	0.60	1.34	0.42	0.67	0.15	0.65	0.81	99.95	0.04
Sw18	1.75	0.89	1.07	0.55	1.35	0.81	0.72	0.56	1.39	0.58	0.70	0.33	0.73	0.84	98.73	0.14
Sw19	2.19	0.64	0.91	0.48	1.44	0.75	0.72	1.30	1.39	0.54	0.69	0.13	0.83	1.03	96.69	2.72
Sw20	1.88	0.36	0.68	0.53	1.38	0.53	0.83	0.54	1.21	0.44	0.60	0.15	0.82	1.00	98.42	1.37
Sw21	1.78	0.39	0.70	0.51	1.40	0.62	0.77	0.85	1.31	0.47	0.65	0.12	0.86	1.02	97.51	1.95
Sw22	1.88	0.37	0.68	0.47	1.45	0.44	0.89	0.46	1.13	0.39	0.56	0.15	0.86	1.18	96.92	1.74

a Not selected for prioritization.

All the Sub-watershed has a low value of mean bifurcation ratio ranging from 1.58 to 3.67. Only sw2 has the highest value i.e., 10.34 which signifies low permeability and high erosion of sw2 [10].

2. Areal parameters:

Table 8 shows low stream frequency (Fs) ranging from 0.43 to 0.83. Low drainage density (Dd) suggests a very coarse texture of Sub-watersheds [27]. The constant of channel maintenance (Ccm) suggests the least erodible nature of sub-watersheds based on Schumm's classification [28]. The infiltration number (If) value ranges from 0.39 (sw22, least erosive) to 0.74 (sw8, most erosive) [14]. Length of overland flow (Lo) value ranges from 0.44 to 0.71; sw1 being the least erodible and sw12 and sw13 as the most erodible Sub-watersheds. The form factor (Rf) value ranges from 0.10 to 0.89 indicating an elongated shape except for sw18 which is circular [29]. The elongation ratio indicates sw14 and sw18 as circular shapes; sw16 and sw15 are oval; and other Sub-watersheds are elongated in shape [30]. The circularity ratio (Rc) value ranges from 0.15 to 0.62 indicating sw17 is the most circular whereas sw1 is the most elongated [10]. Similarly, all the Sub-watershed are in the youth stage except sw17 which is in the mature stage of topography [11]. The 1.27 to 2.61 value of gravelius compactness coefficient (*Cc*) suggests sw17 as the most elongated Sub-watershed and erosive [27].

3. Relief parameters:

Table 8 shows relief ratio (Rr) value ranges from 0.002 to 0.33; sw18 with high relief and steep slope, indicating the most erosive nature [30]. Similarly, the dissection index (DI) value ranges from 0.23 to 0.92 indicating sw8 as being the least erosive and sw7 and sw3 as being the most erosive nature [14]. The ruggedness number (Rn) ranges from 0.05 to 1.60 indicating sw8, sw4, sw9, sw13, sw17, sw14, sw18, and sw16 has low Rn value and hence are less vulnerable to soil erosion while other Sub-watersheds have higher values with sw7 being more vulnerable to erosion [31].

4. Landuse landcover

The landuse landcover was represented as percentage coverage to each Sub-watershed. Table 8 shows the percentage area of merged Lulc classes for each Sub-watershed.

### 3.2 Principal Component Analysis

KMO and Bartlett's Test of sphericity value 0.63 (i.e., >0.5) and significant value of 3.1165E-90 (i.e., <0.05) as shown in Table 5 suggested that PCA can be done [32].

**Table 5:** KMO and Bartlett's test.

Kaiser-Meyer-Olkin Measure of Sampling Adequacy.		.634
Bartlett's Test of Sphericity	Approx. Chi-Square	708.324
	df	105
	Sig.	3.1165E-90

Intercorrelation among the geomorphic parameters: The correlation matrix of selected 15 geometric and landuse parameters was obtained using SPSS statistics 23 software. The correlation coefficient >0.9 suggests the parameter is strongly correlated, the correlation coefficient >0.75 suggest good and the correlation coefficient >0.6 suggest a moderate correlation.

**Fig. 5:**
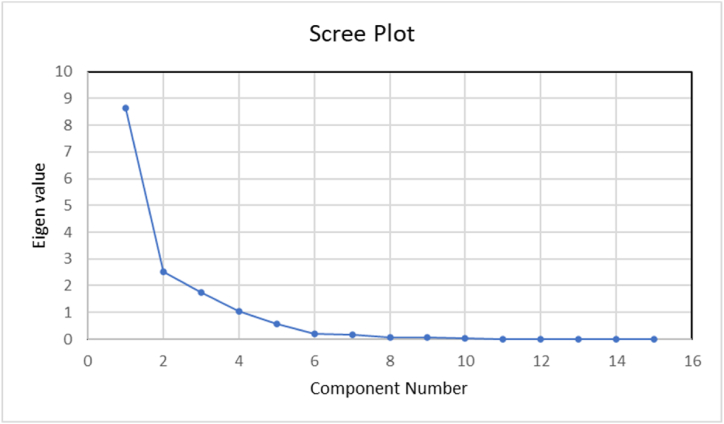
Scree plot.

Total variance or first-factor loading: PCA application on the selected parameters resulted in 4 principal components (PCs) Fig. 5. These components explained about 92.858% of total variance with Eigenvalues greater than 1 Table 6.

**Table 6:** Total variance explained.

Component	Initial Eigenvalues			Extraction Sums of Squared Loadings			Rotation Sums of Squared Loadings		
	Total	% of Variance	Cumulative %	Total	% of Variance	Cumulative %	Total	% of Variance	Cumulative %
1	8.636	57.574	57.574	8.636	57.574	57.574	7.551	50.342	50.342
2	2.510	16.732	74.306	2.510	16.732	74.306	2.616	17.437	67.779
3	1.732	11.547	85.853	1.732	11.547	85.853	2.183	14.553	82.331
4	1.051	7.005	92.858	1.051	7.005	92.858	1.579	10.527	92.858
5	.569	3.794	96.652						
6	.205	1.369	98.021						
7	.153	1.023	99.044						
8	.061	.404	99.448						
9	.058	.389	99.837						
10	.016	.109	99.946						
11	.005	.032	99.978						
12	.002	.015	99.993						
13	.001	.005	99.998						
14	.000	.001	99.999						
15	8.116E-05	.001	100.000						

Extraction Method: Principal Component Analysis.

The correlation explained that some of the parameters have a strong correlation, some have a good and some have a moderate correlation, while some do not correlate at all with any of the components. So, the rotated matrix is necessary to identify the significant parameters.

Rotation of 1st factor loading matrix: The rotated factor loading (Table 7) shows that the first component has a strong correlation with Rf, Rc, Re, and *Cc*; the second component has a strong correlation with Rbm; the third component has a strong correlation with Rn and DI; and fourth component has good correlation with Fs. Thus, Re, Rbm, Rn, and Fs were selected as the most important parameters for watershed prioritization.

**Table 7:** Unrotated and Rotated component matrix.

Component Matrixa					Rotated Component Matrixa				
	Component					Component			
	1	2	3	4		1	2	3	4
Rbm	−.273	.138	0.83**	−.329	Rbm	.056	0.92*	.184	.064
Rf	0.84**	.308	.292	−.108	Rf	.928	.032	−.028	.196
Re	0.87**	.277	.291	−.157	Re	0.96*	.035	−.019	.141
Rc	0.91*	.055	.123	−.312	Rc	.953	−.089	.011	−.153
*Cc*	−0.89**	−.043	−.188	.321	*Cc*	−.949	.030	−.049	.148
Fs	.393	0.66***	.251	.576	Fs	.397	−.059	−.127	0.90**
Dd	−0.94*	−.045	.105	−.173	Dd	−.807	.505	−.075	−.148
Ccm	0.94*	.086	−.109	.182	Ccm	.808	−.501	.044	.177
If	−.594	.594	.364	.350	If	−.439	.487	−.203	.700
Lo	0.94*	.097	−.106	.185	Lo	.813	−.498	.038	.187
DI	.135	−0.81**	.467	.275	DI	.022	−.029	.975	−.102
Rr	0.86**	.128	.097	−.030	Rr	.844	−.204	.036	.097
Rn	.099	−0.78**	.547	.253	Rn	.019	.063	0.98*	−.079
tree	0.89**	−.331	−.188	.076	tree	.692	−.597	.278	−.165
crops	−0.87**	.352	.195	−.086	crops	−.663	.603	−.291	.171
a. 4 components extracted.					a. Rotation converged in 5 iterations.				

*Strong correlation (r > 0.9), ** Good correlation (0.90≥ r > 0.75), *** Moderate correlation (0.75 ≥ r > 0.60).

Preliminary ranking of significant parameters: Preliminary ranking of significant parameters was done based on their relationship with erodibility. Rbm, Rn, and Fs have a direct relationship with erodibility and Re has an indirect relationship.

### 3.3 Weighted Sum Analysis (WSA)

Wsp was calculated using equation (1). Table 8 shows an intercorrelation matrix of significant parameters and weighted sum parameters of different parameters.

**Table 8:** Intercorrelation matrix of Significant parameters.

	Re	Rbm	Rn	Fs
Re	1.000	.046	−.005	.491
Rbm	.046	1.000	.219	.027
Rn	−.005	.219	1.000	−.194
Fs	.491	.027	−.194	1.000
sum	1.533	1.292	1.019	1.324
grand total	5.168	5.168	5.168	5.168
WSA	0.297	0.250	0.197	0.256

### 3.4 .Prioritization of sub-watershed using PCA-WSA

**Fig. 6:**
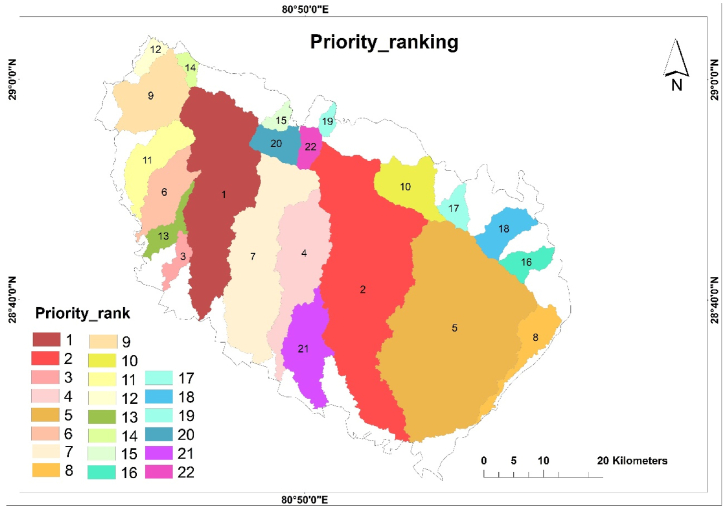
Priority ranking map of Sub-watersheds.

Table 9 shows the preliminary ranking, CV values, and final priority ranking based on the Wsp and PRsp for each Sub-watershed. CV value was calculated using equation (2). The Sub-watershed having the lowest CV value i.e., sw7 was ranked 1 whereas the Sub-watershed having the highest value i.e., sw17 was ranked 22 (Fig. 6).

**Table 9:** Priority ranking of Sub-Watersheds.

sub-watershed	Preliminary ranking				sub-watershed	Final ranking				CV	priority ranking
	Re rank	Rbm rank	Rn rank	Fs rank		Re rank	Rbm rank	Rn rank	Fs rank		
sw1	1.0	10.50	10.00	20.00	sw1	0.3	2.63	1.97	5.12	10.02	8
sw2	17.0	1.00	3.00	11.00	sw2	5.0	0.25	0.59	2.82	8.70	5
sw3	4.5	9.00	4.00	10.00	sw3	1.3	2.25	0.79	2.56	6.94	2
sw4	9.5	18.00	21.00	18.50	sw4	2.8	4.50	4.14	4.74	16.20	21
sw5	3.0	16.00	6.00	8.50	sw5	0.9	4.00	1.18	2.18	8.25	4
sw6	6.5	10.50	5.00	13.50	sw6	1.9	2.63	0.99	3.46	9.00	7
sw7	6.5	3.00	1.00	12.00	sw7	1.9	0.75	0.20	3.07	5.95	1
sw8	4.5	2.00	22.00	5.50	sw8	1.3	0.50	4.34	1.41	7.58	3
sw9	2.0	14.50	20.00	15.00	sw9	0.6	3.63	3.94	3.84	12.01	13
sw10	8.0	4.00	7.00	16.00	sw10	2.4	1.00	1.38	4.10	8.85	6
sw11	9.5	8.00	2.00	22.00	sw11	2.8	2.00	0.39	5.64	10.85	11
sw12	15.0	7.00	8.50	8.50	sw12	4.4	1.75	1.68	2.18	10.05	9
sw13	11.0	14.50	19.00	1.00	sw13	3.3	3.63	3.75	0.26	10.89	12
sw14	21.0	5.00	17.50	5.50	sw14	6.2	1.25	3.45	1.41	12.34	14
sw15	19.0	19.50	11.00	3.00	sw15	5.6	4.88	2.17	0.77	13.45	15
sw16	18.0	22.00	15.00	7.00	sw16	5.3	5.50	2.96	1.79	15.59	20
sw17	16.0	21.00	17.50	17.00	sw17	4.7	5.25	3.45	4.36	17.80	22
sw18	22.0	19.50	16.00	2.00	sw18	6.5	4.88	3.16	0.51	15.07	19
sw19	20.0	6.00	12.00	4.00	sw19	5.9	1.50	2.37	1.02	10.82	10
sw20	12.5	12.50	14.00	18.50	sw20	3.7	3.13	2.76	4.74	14.33	17
sw21	14.0	17.00	13.00	13.50	sw21	4.2	4.25	2.56	3.46	14.42	18
sw22	12.5	12.50	8.50	21.00	sw22	3.7	3.13	1.68	5.38	13.89	16

**Fig. 7:**
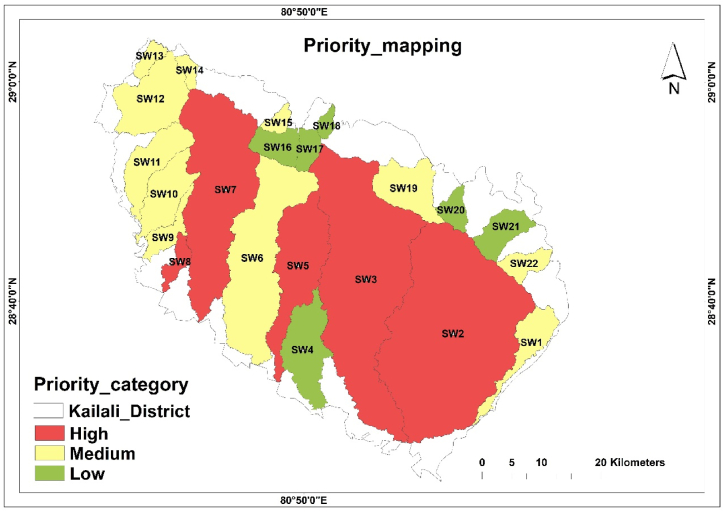
Priority category mapping.

The final priority ranking suggests that sw7 is at the highest risk of erosion whereas sw17 is at the lowest risk of erosion. Those with the highest ranking require the highest priority for soil conservation measures than a medium and low priority. Similarly, about 163511.22 ha (i.e., 61.58%) of Sub-watersheds fall under high priority categories, whereas about 77390.13 ha (i.e., 29.15%) fall under medium categories and about 24603.85 ha (i.e., 9.27%) falls under low priority categories (Table 10; Fig. 7).

**Table 10:** Priority category.

S·N.	CV value	Priority Category	Sub-Watershed	Area (ha)	Area%
1	<8.85	High	SW7, SW3, SW8, SW5, SW2	163511.22	61.58
2	8.85–14.32	Medium	SW10, SW6, SW1, SW12, SW19, SW11, SW13, SW9, SW14, SW15, SW22	77390.13	29.15
3	>14.32	Low	SW20, SW21, SW18, SW16, SW4, SW17	24603.85	9.27

### 3.5 Data quality issue

This study uses O'Callaghan and Mark models with ALOS DEM of 30 *m* resolution for basin and drainage network extractions. The resulting drainage network doesn't lie along the natural river network in some parts of southern flat areas. Similar issues of undefined flow during automated extraction of drainage networks in flat areas using DEM are also discussed by Ref. [33]. These issues are unavoidable because flat areas are connected regions with no internal gradients and the depression filling process in DEM actually increases the size and number of flat surfaces in DEM [34]. Therefore, the basins and streams network derived from DEM may not agree with ground features. Different algorithms have been generated by authors [35,36] to reduce this issue to some extent.

### 3.6 Comparison of approaches of prioritization

Three approaches of prioritization i.e., without PCA or WSA (general method), with PCA only (PCA method), and using PCA-WSA approaches were compared. CV values for the general method were calculated by taking the average of the selected parameter's rankings and CV values for the PCA method were calculated by taking an average of the significant parameter's ranking. Similarly, the priority ranking for each method was calculated based on CV values. When sub-watersheds were ranked using the general method, different sub-watersheds were found to have a common ranking. When ranked using PCA only, sw1 and sw11 as well as sw20 and sw21 were found to have a common ranking and when ranked using PCA-WSA approaches unique ranks were observed as shown in Table 11.

**Table 11:** Comparative approaches of priority ranking.

Sub-watershed	General method priority ranking	PCA method priority ranking	PCA-WSA priority ranking
sw1	7	9.5	8
sw2	3	3	5
sw3	3	2	2
sw4	14.5	21	21
sw5	1	4	4
sw6	8	7	7
sw7	5	1	1
sw8	6	5	3
sw9	9	14	13
sw10	3	6	6
sw11	10	9.5	11
sw12	17.8	8	9
sw13	19	12	12
sw14	14.5	13	14
sw15	13	15	15
sw16	21	20	20
sw17	22	22	22
sw18	20	19	19
sw19	11	11	10
sw20	17.8	17.5	17
sw21	16	17.5	18
sw22	12	16	16

## 4 Conclusion

Watershed prioritization plays a key role in soil and watershed conservation. It helps in the identification and ranking of different degraded watersheds or sub-watersheds into different risk categories which can be used to prioritize the conservation treatments and budgets effectively. This study was conducted for the prioritization of watersheds of the Kailali district in terms of erosion risk. ALOS DEM and LULC maps were used for the calculation of morphometric and Lulc parameters. An integrated PCA-WSA approach was used for prioritization, where PCA was used to calculate the significant parameters and WSA was used in ranking these parameters based on CV values. Elongation ratio (Re), Mean bifurcation ratio (Rbm), and Stream frequency (Fs) were found to be significant parameters. Sub-watershed 7 was found to be at the highest risk of erosion whereas Sub-watershed 17 was at the lowest risk of erosion. About 61.58% of the area of Sub-watersheds falls under high-priority categories, 29.15% of the area falls under medium categories and 9.27% of the area falls under low-priority categories. Different land rehabilitation programs and bioengineering techniques should be focused on the Sub-watershed of high-priority categories followed by medium and low-priority categories to control further soil erosion.

A comparison of different approaches of prioritization was also performed to identify their effectiveness in prioritization. The integrated approaches of PCA-WSA were found to be more effective than the general methods, and the method used PCA only. Future studies can be performed for other issues such as flash floods, groundwater potentiality, landslide susceptibility, and drought using the adopted methodology.

## Author contribution statement

Susil Ojha: conceived and designed the experiments; performed the experiments; analyzed and interpreted the data; contributed reagents, materials, analysis tools or data; wrote the paper.

Lila Puri: conceived and designed the experiments; analyzed and interpreted the data.

Suraj Prasad Bist, Arjun Prasad Bastola, Bishwabandhu Acharya: analyzed and interpreted the data; wrote the paper.

## Funding statement

This work was supported by Tribhuvan University.

## Declaration of competing interest

The authors declare no conflict of interest.
